# The required absolute PASI score to achieve DLQI remission

**DOI:** 10.1111/1346-8138.17723

**Published:** 2025-03-26

**Authors:** Emi Nishida, Akimichi Morita

**Affiliations:** ^1^ Department of Dermatology Nagoya City University West Medical Center Nagoya Japan; ^2^ Department of Geriatric and Environmental Dermatology Nagoya City University, Graduate School of Medical Sciences Nagoya Japan

**Keywords:** absolute PASI, DLQI, PASI, psoriasis

## Abstract

Psoriasis is a chronic inflammatory skin disease that significantly impacts patients' quality of life (QoL). While the Psoriasis Area and Severity Index (PASI) has traditionally been used to assess disease severity and treatment response, achieving substantial improvement in QoL has become an increasingly important therapeutic goal. Recent advances in biologic therapies have enabled higher rates of PASI 90 and PASI 100 responses; however, PASI 75 is no longer considered sufficient for optimal patient outcomes. Calculating PASI improvement rates in daily practice remains challenging, shifting focus toward absolute PASI values as a practical indicator of disease control. In this study, we analyzed 235 psoriasis patients treated with biologics at Nagoya City University Hospital, evaluating 3526 data points collected over a maximum of 288 weeks. We found that PASI positively correlated with DLQI (*r* = 0.5696, *p* < 0.001), and that an absolute PASI score of ≤2.2 was associated with DLQI remission (AUC = 0.8140). Notably, 82.6% of patients achieving PASI 100 also achieved DLQI 0/1 status. However, 1.0% of patients with a PASI score of 0 still reported a DLQI ≥10, suggesting that factors beyond skin lesions, such as stigma or residual damage, may contribute to impaired QoL. These findings underscore the importance of evaluating absolute PASI values to guide treatment decisions and achieve DLQI remission. Additionally, the psychosocial burden of psoriasis must be addressed to ensure comprehensive care and sustained improvements in QoL.

Psoriasis is a chronic skin disease that is often difficult to treat. Individuals with psoriasis often experience discomfort from their skin condition and related symptoms, which not only affect their interactions and environment but also significantly diminish their overall quality of life (QoL). The Psoriasis Area and Severity Index (PASI) score is an assessment tool for psoriasis that is commonly employed in daily practice to evaluate the skin symptoms of psoriasis patients. The PASI improvement rate is used to measure the improvement in psoriasis and set treatment goals for the condition. Several biologic therapies were recently developed, with more than 80% of patients achieving a PASI 90 response and nearly 60% achieving a PASI 100 response. Achieving PASI 75, which represents significant symptom reduction, was once the standard for treatment success, but this level of improvement may not substantially enhance the patient's overall QoL, therefore the treatment goal has shifted from PASI 75 to PASI 90 and PASI clearance. Calculating the PASI improvement rate based on daily medical examinations, however, is challenging. The complexity of calculating the PASI improvement rate has led to a shift toward using the absolute PASI value, which might be a better indicator regardless of the baseline PASI levels.^1^ QoL scores are also gaining attention as an important metric in the management of psoriasis.^2^ Assessment of the absolute PASI value at the initial consultation can be used to guide treatment at an early stage and help patients to achieve a Dermatology QoL Index (DLQI)^3^ 0/1 status (DLQI remission). The DLQI is a 10‐item instrument that measures symptoms (1 item), feelings (1 item), daily activities (2 items), leisure (2 items), work/school (1 item), relationship (2 items), and treatment (1 item), with each item graded on a Likert scale of 0–3 for a maximum score of 30. Understanding whether improvement in skin symptoms leads to improvement in the patient's QoL is crucial, prompting reevaluation of the relationship between the PASI and DLQI. Furthermore, Puig et al.^4^ found that most patients with a PASI of 2 or less achieved PASI 90. Another paper looked at the relationship between absolute and relative PASI treatment targets and health‐related quality of life in psoriasis, and found that absolute PASI could be used as an alternative to PASI response in determining treatment efficacy.^5^

The purpose of this study was to investigate whether the absolute value of PASI is necessary to achieve DLQI remission, therefore we analyzed 235 patients (175 men and 60 women) with psoriasis who were treated with one of four biologics (infliximab, adalimumab, ustekinumab, or secukinumab) at Nagoya City University Hospital, and a total of 3526 data points from pre‐dose to post‐dose (maximum 288 weeks) between 2012 and 2015. Data was collected every 2 weeks from baseline to week 6, and every 8 weeks after week 6. The average number of data collections per patient was 14.9 (1–40). The patients' profiles indicated a median age of onset of 32.3 years (range 1–73 years), a median disease duration of 18.9 years (range 0–64 years), and a median baseline PASI score of 18.2 (range 0–58); the proportion of patients with PsA was 38.3%.

We first found that the PASI score positively correlated with the DLQI (*r* = 0.5696, *P* < 0.001) (Figure 1a). Next we evaluated the receiver operating characteristic curves based on univariate logistic regression. This analysis revealed that a PASI score of 2.2 was needed to achieve a DLQI of 0/1 (DLQI remission, AUC = 0.8140) (Figure S1). On the other hand, 82.6% of patients who achieved PASI 100 also had DLQI 0/1. Even patients with a PASI score of 0 and no skin lesions may have a reduced QoL. Surprisingly, 1.0% (*n* = 8) of patients with a PASI score of 0 had a DLQI score of ≥10 (Figure 1b). This may be attributed to the correlation between DLQI and stigma associated with the disease^6^ or to other residual damage beyond the skin symptoms. Our findings indicate that the absolute PASI values should be <2.2 to consider the patient in DLQI remission.

**FIGURE 1:**
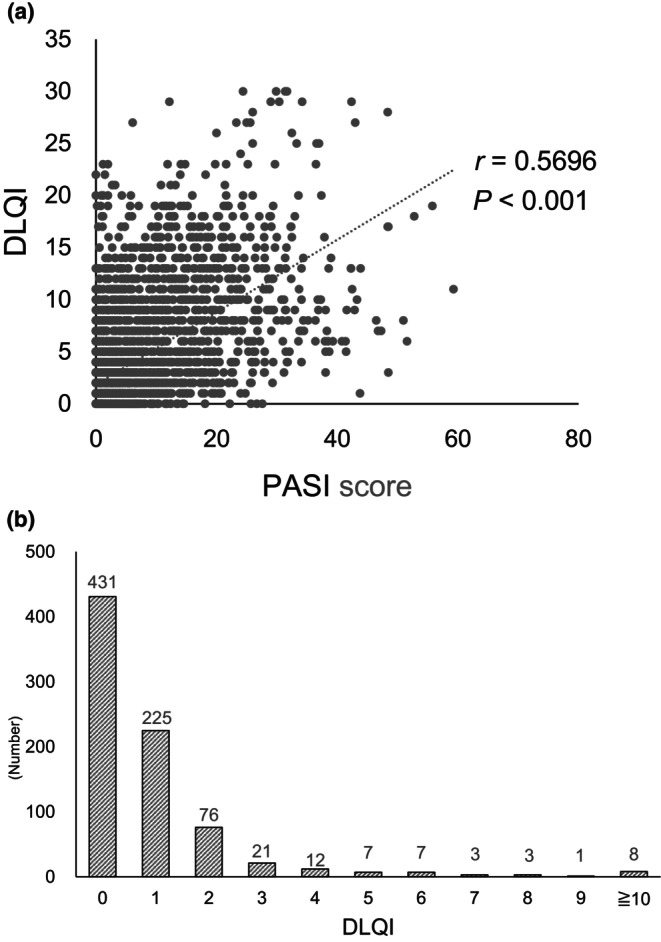
(a) Correlation between the PASI score and DLQI. (b) Number of each DLQI when PASI clear was achieved.

It is important to recognize that psoriasis patients often experience significant stigma related to their condition and their circumstances, and may gradually learn to cope with their disease during treatment. Assessment of the absolute PASI is crucial for achieving DLQI remission.

## CONFLICT OF INTEREST STATEMENT

None declared.

## Supporting information


**Supporting Information Figure S1.** PASI cutoff value to achieve a DLQI reimission(DLQI0/1).
