# Supplementary material for: The required absolute PASI score to achieve DLQI remission
Source: J Dermatol. 2025 Mar 26;52(5):947–9. doi: 10.1111/1346-8138.17723 (PMC12056283; doi:10.1111/1346-8138.17723)
Supplement: Supplementary file 1 — Supporting Information Figure S1. PASI cutoff value to achieve a DLQI reimission(DLQI0/1). [file JDE-52-947-s001.docx]

**Supplementary Material**

Figure legend

PASI cut off value to achieve a DLQI remission(DLQI 0/1)

Cut off value=2.2 (sensitivity/ specificity 0.268/0.759)
